# Pediatric rheumatology infusion center: report on therapeutic protocols and infusions given over 4 Years with focus on adverse events over 1 Year

**DOI:** 10.1186/s12969-018-0234-0

**Published:** 2018-03-09

**Authors:** Surabhi S. Vinod, Annelle B. Reed, Jamelle Maxwell, Randy Q. Cron, Matthew L. Stoll

**Affiliations:** 1 Department of Pediatrics, University of Alabama School of Medicine, Birmingham, AL USA; 2 Division of Pediatric Rheumatology, Children’s of Alabama, Birmingham, AL 35233-1711 USA

**Keywords:** Infusion center, Adverse reaction, Therapeutic protocols, Infliximab, Methylprednisolone, Rituximab

## Abstract

**Background:**

Children with chronic rheumatic disease often require intravenous (IV) therapy. Our center has instituted standardized protocols for use of IV medications in rheumatology patients. Herein, we introduce the therapeutic protocols and report on their short-term safety.

**Methods:**

This was an institutional review board (IRB) approved retrospective chart review of all patients who had received IV infusions between the years 2012 and 2015 at a single center, prescribed by a pediatric rheumatologist. Infusion medications included abatacept, belimumab, cyclophosphamide, immune globulin, infliximab, methylprednisolone, N-acetylcysteine, pamidronate disodium, rituximab, and tocilizumab. For calendar year 2015, all adverse infusions reactions were recorded along with treatment strategies used to manage them, and outcomes. Rates of adverse events were calculated per infusion medication.

**Results:**

During calendar years 2012–2015, 7585 IV infusions were administered to 398 unique patients. In the year 2015, 2187 infusions were administered to 224 patients, with 34 patients experiencing 41 infusion reactions (1.9% of all infusions). Rituximab had the highest rate of adverse drug reactions with 10 patients experiencing reactions during 106 infusions (9.4%). None of the reactions were life-threatening, and only 6 resulted in discontinuation of therapy.

**Conclusions:**

In a recent 4-year span, the UAB Pediatric Rheumatology Infusion Center has given thousands of IV infusions with minimal adverse reactions over a one-year reporting period. The combination of standard infusion protocols, experience of and communication between physicians and nurses who staff the center, and safety of the medications themselves, allows for safe IV administration of a variety of therapies for pediatric rheumatology patients.

**Trial registration:**

Not applicable; this was a retrospective study.

**Electronic supplementary material:**

The online version of this article (10.1186/s12969-018-0234-0) contains supplementary material, which is available to authorized users.

## Background

Intravenous (IV) infusion therapy is now critical for the treatment and maintenance of many pediatric rheumatic diseases, such as juvenile idiopathic arthritis (JIA), systemic lupus erythematosus (SLE), and inflammatory bowel disease (IBD) associated arthritis. Prior to biologic infusion therapy, non-steroidal anti-inflammatory drugs (NSAIDs), corticosteroids (CS), and non-biologic disease modifying anti-rheumatic drugs (DMARDs) were a mainstay of treatment [1, 2]. However, due to the strong adverse event (AE) profile of CS and the limited benefit to side effect ratio of NSAIDs, these drugs are often now used as a means of bridging therapy until better alternatives are used [2]. In this era, biologic therapies have become increasingly used treatment approaches, and have made lasting changes in the quality of life of patients affected by diseases such as JIA [3]. Many of these novel biologic therapies require or at least may be administered as IV infusions. While some patients receive such therapies at home via home health or through local infusion centers, which have advantages of decreased travel burden and in some cases more flexible hours, the majority of families prefer to receive them in a well-equipped infusion center associated with a tertiary hospital, and certain medications (e.g., rituximab) are never administered via home health agencies. Although receipt of the infusions at the tertiary hospital poses some inconvenience for the family, this is mitigated to some extent by scheduling visits on the same day as the infusion, which can take place in the infusion center or in the adjacent rheumatology clinic.

In order to cope with the increased demand for providing IV infusion therapies for children with rheumatic diseases, there has been a need for expanding existing, or creating new, infusion facilities. With the creation of new pediatric rheumatology program at the University of Alabama at Birmingham (UAB) in 2007, a novel pediatric rheumatology infusion clinic (7 beds) was constructed. Protocols were created and modified to provide safe and effective IV infusions for this pediatric rheumatology population. Herein, the breadth of clinical diagnoses treated, the variety of IV therapies provided (from 2012 to 2015), and the associated AEs (2015) are reported. This information can be used to assist current and future pediatric rheumatology infusion centers in the care of pediatric rheumatology patients requiring a variety of repeated IV infusion therapies.

## Methods

### Overview of study

This is an IRB approved retrospective chart review of all patients given infusions as ordered by UAB pediatric rheumatology providers (6 physicians and 3 nurse practitioners) in the Pediatric Rheumatology Infusion Center from 2012 to 2015, including medications used off-label for the given indications. The Pediatric Rheumatology Infusion Center at UAB operates Monday-Friday from 8:00 am to 4:30 pm with infusions, depending on the medication and dose, lasting between two and 8 h (Table 1). The medications infused include abatacept, belimumab, cyclophosphamide, immune globulin, infliximab, methylprednisolone, N-acetylcysteine, pamidronate disodium, rituximab, and tocilizumab, which were given as outlined by developed standardized protocols (see Additional file 1). The protocols include pre-medications to prevent allergic reactions as well as rescue medicines should allergic-type reactions occur.

**Table 1:** Duration of infusions

Medications by Generic Name	Duration of Infusion
Abatacept	2 h
Belimumab	2 h
Cyclophosphamide	6–8 h
Immune Globulin-IVIG	2–8 h
Infliximab	2–6 h
Methylprednisolone	2 h
N-acetyl cysteine	5 h
Pamidronate	4–6 h
Rituximab	6-8 h
Tocilizumab	2–4 h

### Data collection

Clinical data about the infusion patients was collected directly from the electronic medical record (EMR). This data included diagnosis, gender, infusion medications given, age at first and last infusions, and number of infusions given per patient. For calendar year 2015, a hand-search of each infusion was performed to identify any AEs that occurred during the infusions, as well as management of the reactions. All infusions are documented in the EMR. Every 15 min during the first hour of the infusion, every 30 min during the second hour of the infusion, and every 60 min thereafter, the nursing staff responsible for the infusion documents the patient’s vital signs, response to therapy, rate changes, and IV site condition. Adverse drug events, as well as phone calls made and actions taken, are documented in the EMR as well. Safety events that occurred outside of the infusions (e.g., infections) were not collected as part of this report.

### Statistical analysis

Comparisons of dichotomous variables were performed with the Chi-squared or Fisher exact tests, as appropriate; comparisons of continuous variables were performed with the Student’s t-test. Microsoft Excel and SPSS (Version 25) were used for analyses of the data.

## Results

From 2012 through 2015, a total of 7585 IV infusions were given to 398 patients in the UAB Pediatric Rheumatology Infusion Center. During the calendar year 2015 alone, a total of 2187 infusions were given to 224 patients. Demographic and clinical data on the patients are shown in Table 2, and a summary of the medications administered by diagnosis is included in Table 3. Among those, thirty-four patients experienced 41 infusion reactions, for a rate of infusion reactions of 1.9% of all infusions (Table 4.) The most common infusion reactions, occurring in 17 patients in total, were nausea/vomiting and throat discomfort, including tightness, itching, or pain. Medications with infusion reactions were abatacept, infliximab, immune globulin, methylprednisolone, rituximab, and tocilizumab. Rituximab had the highest rate of adverse drug reactions with 10 events over 106 infusions (9.4%), while belimumab, cyclophosphamide, and pamidronate were not associated with any. All but one of these reactions terminated with conservative therapy (e.g., non-steroidal anti-inflammatory drugs, diphenhydramine, IV fluids); flushing (rash) following infusion with abatacept was treated with subcutaneous epinephrine. All of the drug-specific protocols have nursing instructions in the event of any infusion reaction (see Additional file 1). Following those protocols, all reactions were managed by placing the infusion on hold and giving established treatments. Specifically, pain was treated with ibuprofen or acetaminophen; rashes, itching, and flushing were managed with diphenhydramine; and nausea with or without emesis were treated were ondansetron or promethazine. As per protocol, if the symptoms were deemed non-life threatening and resolved with conservative therapy as above, re-starting of the infusion was permitted. Infliximab and rituximab were often re-started at slower rates, which were gradually increased as tolerated. Only six of these infusion reactions required discontinuation of therapy (infliximab, *n* = 3; methylprednisolone, *n* = 2; rituximab, *n* = 1); the patient who received epinephrine for suspected anaphylactic reaction to abatacept successfully tolerated additional doses. Overall, of the 224 patients who received infusions in 2015, 139 continued to receive infusions at our center in 2016, 19 transferred care due to moving out of state or graduating to adult care, 11 switched to a more convenient location or method of delivery, and 33 discontinued due to disease control. Only 6 discontinued due to adverse events, 5 due to parental choice, and 21 due to inefficacy, of whom 10 successfully switched to a different infusion.

**Table 2:** Demographic and clinical features of the patient population

Feature	*n* (224)
DIAGNOSIS	
Juvenile idiopathic arthritis	100
RF-negative polyarticular	35
Enthesitis-related arthritis	24
Oligoarticular	17
Psoriatic	12
Systemic	7
RF+ polyarticular	5
Systemic lupus erythematosus	40
Inflammatory bowel disease-associated arthritis	18
Juvenile dermatomyositis	12
Idiopathic uveitis	10
Sjogren syndrome	8
Mixed connective tissue disease	7
Henoch-Schonlein purpura	5
Sarcoidosis	4
Chronic recurrent multifocal osteomyelitis	3
Other^1^	14
DEMOGRAPHICS	
Female sex	161 (72%)
Age at initiation of infusion (years: mean ± SD)	11.5 ± 4.2
Age in 2015^2^ (years: mean ± SD)	13.3 ± 4.0
THERAPY	
Therapy duration (start – September 30, 2017); years: mean ± SD	2.8 ± 2.4
Use of antimetabolites^3^	183 (82%)
Outcome of infusions used in 2015	
Continued into 2016	139^4^
Patient transferred care	19
Changed to home/local infusions or subcutaneous administration	11
Stopped due to disease control	33
Stopped due to inefficacy	21^4^
Stopped due to adverse events	6
Stopped as per parental choice	5

^1^The following diagnoses had 1 patient each: Behcet Syndrome, *CREST* (Calcinosis, Raynaud, Esophageal dysmotility, Sclerodactyly, Telangiectasia) syndrome, cutis laxa with restrictive lung disease, eosinophilic granulomatosis with polyangiitis, hyper IgD syndrome, idiopathic pulmonary hemosiderosis, immune-mediated glomerulonephritis, idiopathic thrombocytopenic purpura, mucolipidosis type IV, orbital pseudotumor, pemphigoid, primary angiitis of the central nervous system, polymyositis, relapsing polychondritis. ^2^Calculated as age mid-year (June 30, 2015). ^3^azathioprine, cyclophosphamide, leflunomide, methotrexate, mycophenolate mofetil. ^4^Ten patients were counted in both rows, due to switching from one infusion to another in 2015

**Table 3:** List of infusions by diagnosis

Disease	ABT	Belimumab	CYT	IVIg	INX	MP	Pamidronate	RTX	TCZ
JIA	29		1	1	57	32	1	1	12
SLE		2	14	8		35		22	
IBD-a					17	5			
JDM				11		11			
Uveitis					9	2		1	
Sjogren						8		7	
MCTD	2			1	1	6		3	
HSP			1	3		5		3	
Sarcoidosis					4	3			
CRMO					2	1	1		
GPA						3		3	
Other			1	6	4	9	1	5	
Total patients	31	2	17	30	94	120	3	45	12
Total infusions	237	26	76	266	816	513	7	106	140

Abbreviations: *ABT* Abatacept, *CRMO* Chronic recurrent multifocal osteomyelitis, *GPA* Granulomatosis with polyangiitis, *HSP* Henoch-Schonlein purpura, *IBD-a* Inflammatory bowel disease-associated arthritis; *INX* Infliximab, *IVIg* Intravenous immunoglobulin, *JDM* Juvenile dermatomyositis, *JIA* Juvenile idiopathic arthritis, *MCTD* Mixed connective tissue disease, *MP* Methylprednisolone, *RTX* Rituximab, *SLE* Systemic lupus erythematosus, *TCZ* Tocilizumab

**Table 4:** Number of infusion reactions in 2015

Reaction	ABT (237)	IVIG (266)	INX (816)	MP (513)	RTX (106)	TCZ (140)	Total Infusion Reactions
Rash	1	2			1		4
Nausea/Vomiting		2	1	5		1	9
Cough			1		2		3
Throat Tightness/Itching/Pain	1	2			5		8
Chest Pain/Tightness		1	1				2
Headache	3	2	3				8
Swelling			2		1		3
Wheezing				1			1
Hives					1		1
Blurry Vision				1			1
Abdominal Pain			1				1
Total Events	5	9	9	7	10	1	41

Medications not associated with any infusion reactions are not included in the table

*Abbreviations*: *ABT* Abatacept, *INX* Infliximab, *IVIG* Intravenous immunoglobulin, *MP* Methylprednisolone, *RTX* Rituximab, *TCZ* Tocilizumab

Of the 224 patients receiving IV infusions, 183 (82%) received one or more anti-metabolites (azathioprine, cyclophosphamide, leflunomide, methotrexate, mycophenolate mofetil). Among the medications as a whole, there was no obvious association between development of infusion reactions and use of an anti-metabolite. Infusion reactions were observed in 9/41 (22%) of patients not taking an anti-metabolite versus 25/183 (14%) of patients on one (*p* = 0.181). However, among patients taking infliximab, there was a trend towards an association, with infusion reactions observed in 3/12 (25%) of patients not taking an anti-metabolite versus 6 / 82 (7.3%, *p* = 0.087, Fisher’s exact test) among patients who were taking an anti-metabolite. Interestingly, among those taking infliximab, patients with an infusion reaction were younger (10 ± 3.8 versus 14 ± 3.8 years, *p* = 0.008, Student’s t-test) than those who did not; this association did not hold for the group as a whole (data not shown). Female patients (29/161, 18%) may have been more likely to develop infusion reactions than males (5/63, 7.9%, *p* = 0.059).

## Discussion

Modern therapeutics have dramatically altered the landscape for children with a variety of rheumatic diseases, including JIA and lupus [3, 4]. Although many biologics have the convenience of injectable delivery that the patient or family can self-administer, still others require IV infusions. In addition to the well-known risks associated with immunosuppressive therapy, in general [5], IV medications have their attendant risks of infusion reactions, that while unusual, can in very rare cases prove life-threatening [6]. With medications such as rituximab and infliximab, it is estimated that up to 10% of adults have an infusion reaction severe enough to warrant permanent discontinuation of therapy [7–9]. There is mixed data as to the risk of infusion reactions in the pediatric population. Aeschlimann et al. [10] reported infusion reactions in only 46/2446 (2%) of infliximab infusions, of which only six resulted in discontinuation. However, Al-Mayouf et al. [11] reported 10 suspected anaphylaxis reactions, 8 of which were associated with infliximab among 52 patients taking infusions. Likewise, Lahdenne et al. [12] reported 12 infusion reactions, 8 of which were deemed severe, among 64 patients taking infliximab despite 100% use of anti-metabolites. In contrast to these experiences, our experience has been largely positive. As a whole, we administered 7585 IV infusions to 398 unique patients from 2012 to 2015. In 2015 alone, 2187 infusions were given to 224 patients. Of those, 41 (1.9%) were associated with an infusion reaction of any sort. However, only 1 reaction was treated with an emergency medication, and only 6 of those 2187 infusion reactions (0.27%) resulted in discontinuation of therapy. All of the reactions that did occur in 2015 would fall under the heading of mild adverse reactions, as defined by the Rheumatology Common Toxicity criteria (RCTC) [13].

Several factors may be responsible for our success with the administration of IV therapy. First, we have a skilled team of medical professionals overseeing the delivery of the infusions. All nurses working in the Infusion Center graduated from an accredited school of nursing with an RN license, possess at least one year of recent nursing experience upon hiring, are certificated in Basic Life Support, and are certificated for the administration of chemotherapy within six months of securing employment. Second, the infusion clinic shares space with the rheumatology clinic, and by policy, at least one pediatric rheumatology physician or nurse practitioner must be present on campus at all times during operation of the infusion clinic. This facilitates rapid telephone or in person assessment of the patient. Consequently, symptoms such as flushing or chest pain that could fall anywhere on the spectrum from anxiety to anaphylaxis are rapidly and efficiently assessed by skilled providers. Third, the detailed infusion protocols (see Additional file 1) provide for pre-medications, call for availability of emergency bedside medications, and specify actions taken during both mild as well as potentially life-threatening infusion reactions. For patients receiving rituximab, data from a randomized trial indicated that pre-treatment with 100 mg of methylprednisolone resulted in decreased risk of infusion reactions [14]. With respect to infliximab, the role of pre-medication is less clear. A randomized trial indicated that pre-medication with betamethasone provided no benefit [15], while observational studies have yield mixed evidence with respect to benefits of anti-histamines [10, 12, 16, 17]. Arguably, however, the rarity (9/816) and mild nature of the infusion reactions among patients taking infliximab provide scant impetus to adjust the protocols. Finally, our positive experiences also speak to the general safety of the medications used. Additional factors that may promote the safety of the medications described herein are high doses accompanied by frequent administration of drugs such as infliximab [18] and frequent use of concomitant DMARDs. Eighty-two percent of patients taking any infusion and 88% of our patients on infliximab used an anti-metabolite. With respect to dose, the mean infliximab dose was 13.8 mg/Kg, with none of our patients receiving doses < 6 mg/Kg. This appears to reflect a strong difference between our patients and those reported by Lahdenne et al. [12] , in whom the average total dose was 243 mg ; the article by Al-Mayouf et al. [11] did not discuss medication doses or pre-medications. These factors appear to result in decreased incidence of development of anti-drug antibodies and of infusion reactions in general [19, 20], and use of anti-metabolites appeared to be protective against the development of infusion reactions among patients taking infliximab in our study; levels of anti-drug antibodies were not routinely available in our patients. It is possible that the safety profile described herein is due to the fact that many of the patients had been on therapy for several years (Table 2) and thus, those patients who were prone to infusion reactions may have already discontinued the therapy. However, infusion reactions were no more likely among those patients newly started on therapy in 2015 (11/80, 14%) as compared to those who had started prior to 2015 (23/144, 16%, *p* = 0.657); similar trends were observed among the sub-group taking infliximab (data not shown). Ultimately, the precise factors responsible for the safety of the infusions reported herein cannot be determined, however.

Some of the symptoms reported in the infusion reactions (Table 4) overlap with those that would be seen during life-threatening anaphylactic events, e.g. itching and swelling of the throat and face, hives, rash, and cough, with one episode of nausea/vomiting. The most frequent culprit in our study was rituximab, in which 10 such reactions were observed over 106 infusions (9.4%) given over 2015. A similar experience was reported by Moss et al. [21], in their report of 10 infusions reactions over 185 infusions. In their study, as in ours, all reactions resolved with conservative management, and patients were able to receive subsequent infusions. Similarly, Lequerre et al. [22] reported that instituting a protocol in which similar infusion reactions were treated with slowing the rate of the infusion permitted continuation of therapy in patients who in the past would have had to discontinue treatment with the offending agent. True anaphylactic reactions do not resolve with diphenhydramine, nor would slowing the rate of infusion provide any safety benefit, and repeat infusions would not be tolerated. Therefore, although tryptase levels were not obtained, such events likely represented anaphylactoid reactions. The overlapping symptoms between these reactions and true anaphylaxis underscore the benefits of having an experienced nursing staff as well as immediate availability of trained clinicians. While delays in the treatment of anaphylaxis can be fatal, there are also substantial risks associated with falsely labeling a reaction as anaphylactic, thus permanently depriving the patient of use of a potentially life-saving medication.

## Conclusions

In recent years, the UAB Pediatric Rheumatology Infusion Center has treated a wide variety of diagnoses and given thousands of IV infusions with very few adverse reactions. These reactions were mild and transient, and resolved after using protocol derived management strategies. Together, the use of standardized infusion protocols, the combined experience of physicians and nurses who manage the treatment, effective communication between the nurses providing the infusions and the rheumatology-trained clinicians, and the safety profile of the medications themselves, combine to allow for safe IV infusions for pediatric rheumatology patients, and can serve as a model for the development of future infusion centers.

## Additional file

Additional file 1: Order sets for infusions administered in the pediatric rheumatology clinic at UAB. (PDF 256 kb)

## Abbreviations

AE: Adverse event
CS: Corticosteroids
DMARD: Disease modifying anti-rheumatic drug
EMR: Electronic medical record
IBD: Inflammatory bowel disease
IRB: Institutional review board
IV: Intravenous
JIA: Juvenile idiopathic arthritis
NSAID: Non-steroidal anti-inflammatory drug
OMERACT: Outcome measures in rheumatology
RF: Rheumatoid factor
SLE: Systemic lupus erythematosus
TNF: Tumor necrosis factor
UAB: University of Alabama at Birmingham
