# Supplementary material for: Pediatric rheumatology infusion center: report on therapeutic protocols and infusions given over 4 Years with focus on adverse events over 1 Year
Source: Pediatr Rheumatol Online J. 2018 Mar 9;16:16. doi: 10.1186/s12969-018-0234-0 (PMC5845357; doi:10.1186/s12969-018-0234-0)
Supplement: Supplementary file 1 — Order sets for infusions administered in the pediatric rheumatology clinic at UAB. (PDF 256 kb) [file 12969_2018_234_MOESM1_ESM.pdf]

RHEUMATOLOGY ABATACEPT (Orencia) INFUSION ORDERS # ___

1. Attending MD: ___________________________________
2. Diagnosis: ____________________
3. Weight: ______ kg
4. Diet: Regular
5. Vital Signs: q 15 min x 2, then q 30 min x 2 until infusion complete.
6. Obtain peripheral IV. LMX cream or equivalent prior to insertion PRN.
7. Labs: CBC w diff, ESR, CRP, CMP
8. Medications:
   - PREMEDS: Acetaminophen_________ mg PO (____ mg/kg) PRN
     Diphenhydramine ________ mg IV/PO (_0.5__ mg/kg) PRN
   - ABATACEPT__________ mg (10 mg/kg, max 1000mg) IV diluted in 100 ml normal saline to run over 45 minutes or longer.
9. Stop infusion and notify MD for: SBP less than __90___or greater than ____150___
   DBP less than __50___ or greater than ___90____
   Generalized hives; Fever ≥ 101° F
   Shortness of breath/wheezing;
   Nausea/vomiting/diarrhea
   - Return for next infusion in _______ week/s or abatacept infusion # ____

Signature __________________________/_______________________
Date/Time: ________________

RHEUMATOLOGY: TOCILIZUMAB (Actemra) INFUSION ORDERS # ___

1. Attending MD: ___________________________
2. Diagnosis: ____________________________
3. Weight: Height:______cm BSA:________ BSA(m^2) = $\sqrt{(HT(cm) \times wt (Kg))/3600}$
4. Obtain peripheral IV. LMX cream or equivalent prior to insertion PRN.
5. Labs: CBC w diff, ESR, CRP, CMP, Ferritin, aldolase, D-dimer. triglycerides, total cholesterol with HDL and LDL.
6. Medications.

BEDSIDE MEDS FOR EMERGENCY USE:

Epi-pen Junior (Dose 0.1mg) if patient weight 15-30kg
Epi-pen regular (Dose 0.3mg) if patient weight greater than 30kg
Diphenhydramine ___mg IV (1 mg/kg)
Hydrocortisone ___ mg IV (2 mg/kg)

Tocilizumab _____mg (10 mg/kg, max 1 gram) in __50___ mL NS IV
(note: round total volume up to either 50mL, 100mL, 150 mL, 250mL, or 500mL)

- Vital signs every 15 minutes x 4, then Q 1 hour until infusion completed
- Infuse over 1 hour or longer.

Monitor for signs of adverse reaction: urticaria, diarrhea, arthralgia, headache; dizziness, tachycardia, hypertension, hypotension, fever, vomiting, swelling of lips, tongue, or face, and airway compromise: chest tightness, increased WOB, SOB, wheezing. If symptoms:

1. Stop infusion.
2. Notify MD/PNP of possible reaction.
3. Give diphenhydramine____**mg** (1mg/kg) X 1 dose for rash, redness, or itching.
4. Give Epi-pen IM for airway compromise or reaction involving two body systems (i.e. fever and vomiting, wheezing and rash)

- Return for next infusion in _______ week/s for Actemra # ___

Signature _____________________________/_________________________
Date/Time: ___________________

RHEUMATOLOGY: Belimumab (Benlysta) INFUSION ORDERS # ___

1. Attending MD: ________________________
2. Diagnosis: ___________________________
3. Weight:_______kg
4. Obtain peripheral IV. LMX cream or equivalent prior to insertion PRN.
5. Labs: CBC w diff, ESR, CRP, CMP, urinanalysis, Urine protein:creatine ratio
6. Medications:

BEDSIDE MEDS FOR EMERGENCY USE:

Epi-pen Junior (Dose 0.1mg) if patient weight 15-30kg
Epi-pen regular (Dose 0.3mg) if patient weight greater than 30kg
Diphenhydramine ________ IV (1 mg/kg)
Hydrocortisone __________ IV (2 mg/kg)

- Premed: **Diphenhydramine** ________mg (0.5mg/kg) PO 30 minutes prior to administration

- **Belimumab** ____________mg (10 mg/kg) in 250 mL NS IV

- Vital signs every 15 minutes x 4, then Q 1 hour until infusion completed
- Infuse over 1 hour or longer.

Monitor for signs of adverse reaction: urticaria, diarrhea, arthralgia, and headache; dizziness, tachycardia, hypertension, hypotension, fever, vomiting, swelling of lips, tongue, or face, and airway compromise: chest tightness increased WOB, SOB, wheezing. If symptoms:

5. Stop infusion.
6. Notify MD/PNP of possible reaction.
7. Give diphenhydramine ____ mg (1mg/kg) X 1 dose for rash, redness, or itching.
8. Give Epi-pen IM for airway compromise or reaction involving two body systems (i.e. fever and vomiting, wheezing and rash)

- Return for next infusion in ______ week/s for Belimumab # __

Signature ______________________________/__________________________
Date/Time: ____________________

RHEUMATOLOGY CYCLOPHOSPHAMIDE (Cytoxan) INFUSION ORDERS # ___

Date:___ / ____/ ______ Time:____________________ **Dose: # _____________**

MEASUREMENTS

Admit Wt: ______ **Kg** ☒ Measured **Ht:**_______**Cm** ☒ Measured **BSA (m2):** ________

- Items with boxes must be checked to be ordered
- Strike thru must be thru entire line and must be initialed by the physician to be valid.
- The following abbreviations CANNOT be used: **cc u IU q.d. q.o.d. MS MS04 MgSO4**
- CANNOT use trailing Zero (X.0 mg) or Leading decimal point (.X mg) (always use a leading zero)
- The metric system must be used to enter all medication orders
- All pages of order set MUST be sent to Pharmacy

ALLERGIES/SENSITIVITIES

☐No Known Allergies ☐Latex Allergies ☐Latex Precautions ☐Unable to Obtain

**Agent**	**Reaction(s)/Notes**
1.	
2.	
**Other:**	

INDICATION FOR USE OF THIS ORDER SET – Cyclophosphamide (Cytoxan®)

Indication: **ICD10:**

PRESCRIBER RESTRICTIONS/INFORMATION

Labs to be drawn prior to start of infusion

Negative pregnancy status must be documented prior to infusion

All cyclophosphamide orders must be signed by an attending or fellow physician

VITALS

	**DESCRIPTION**	**FREQUENCY**	**NOTIFY PRESCRIBER**	**INSTRUCTIONS**
☒	Vital Signs (Pulse, Respirations)	☒Every 2 hours during infusion<br>☐ ________	☐ HR greater than ____ or less than _____<br>☐ RR greater than ____ or less than _____	
☒	Temperature	☒Every 2 hours during infusion<br>☐ ________	☐ Temp less than ___<br>☒ Temp greater than _101__	
☒	Blood Pressure	☒Every 2 hours during infusion<br>☐ ________	☒ SBP greater than __150___or less than __90__<br>☒ DBP greater than _90____or less than __50__	

NURSING/TREATMENTS

	**DESCRIPTION**	**FREQUENCY**	**INSTRUCTIONS**
☐	Intake and Output	☐Strict (Measure) ☐Routine (Count)<br>☐Every ______during infusion	Notify Prescriber if UOP drops below 1 mL/kg/hr averaged over ______ hours
☒	Notify Prescriber	☒ For suspected medication reaction	☐If patient loses IV access

	**VASCULAR ACCESS**	**INSTRUCTIONS**	**COMMENTS**
☒	Peripheral IV ☒ Insert ☒ Maintain	Saline lock when not in use	Site care per policy
☐	Central Vascular Access	**See Central Vascular Access Order Set**	

MEDICATIONS FOR VASCULAR ACCESS

	**MEDICATION NAME**	**DOSE**	**ROUTE**	**FREQUENCY**	**INSTRUCTIONS**
☒	Lidocaine 4% Cream (LMX)	1 Application (Dispense 1 tube)	Topical-skin (procedure site)	PRN venipuncture (max every 2 hours)	Apply 30 minutes before procedure with occlusive dressing

Licensed Independent Practitioner Initials (Prescriber) **RN SIGNATURE DATE/TIME**

DT0077

MEDICATION ORDER
CYCLOPHOSPHAMIDE
(CYTOXAN®) INFUSION
NON-ONCOLOGY
USE ONLY

Form#0039

STANDARD ADMINISTRATION TIMES

Daily 0800	Nightly 2100	Every 3 hours 0000-0300-0600-0900-1200-1500-1800-2100
Twice a day 0800-2000	Three times a day 0800-1400-2000	Every 4 hours 0000-0400-0800-1200-1600-2000
Every 12 hours 0800-2000	Four times a day 0800-1200-1600-2000	Every 6 hours 0200-0800-1400-2000
		Every 8 hours 0000-0800-1600

IV FLUIDS (must specify 'Amount To Infuse' OR 'Infusion Rate')

	FLUID TYPE	ADDITIVES	ROUTE	AMOUNT TO INFUSE	INFUSION RATE	INFUSE OVER
**IV Maintenance/Other**						
☒	D5W ½ NS		IV		____ mL/hr (125 $mL/m^2/hr$)	Infuse for one hour prior to cyclophosphamide infusion and 3 hours post

PRE-MEDICATION ORDERS

	MEDICATION NAME (GENERIC PREFERRED)	DEFAULT (REFERENCE)	DOSE	ROUTE	FREQUENCY	DURATION	PRIORITY	INSTRUCTIONS (IF PRN – INDICATION IS REQUIRED)
☒	Ondansetron (Zofran®)	0.45 mg/kg (Max: 24 mg/dose)		IV	ONCE			30 minutes prior to infusion
**CYCLOPHOSPHAMIDE - INFUSION AND ADMINSTRATION INSTRUCTIONS**								
☒	Mesna	30% of cyclophosphamide dose		IV	Every 4 hours	2 doses		1st dose – 15 minutes prior to infusion<br>2nd dose - 3 hours after infusion (Round to nearest 10mg)
☒	Cyclophosphamide (Cytoxan®)	___**750**____mg/m^2		IV	ONCE			Pharmacy note: Dilute in NS<br>**Infuse cyclophosphamide over 1 hour.**

LABS-

	NAME OF TEST	PRIORITY	FREQUENCY	DURATION	INSTRUCTIONS/PRN INDICATIONS
☒	Complete Blood Count with differential	Routine	Once		Obtain prior to start of infusion.
☒	Comprehensive Metabolic Panel	Routine	Once		Obtain prior to start of infusion.
☐	Phosphorus	Routine			Obtain prior to start of infusion.
☒	C-reactive protein	Routine	Once		Obtain prior to start of infusion.
☒	Erythrocyte Sedimentation Rate	Routine	Once		Obtain prior to start of infusion.
☒	Urine pregnancy	Routine	Once		Obtain prior to start of infusion.<br>**Negative pregnancy status must be documented prior to infusion**
☒	Urinalysis	Routine	Once		Obtain prior to start of infusion.
☒	Urine Protein/Creatinine	Routine	Once		Obtain prior to start of infusion.
☒	Complement C3	Routine	Once		Obtain prior to start of infusion.
☒	Complement C4	Routine	Once		Obtain prior to start of infusion.
☒	dsDNA (DNA double strand Antibody level)	Routine	Once		Obtain prior to start of infusion.<br>Reference lab

Licensed Independent Practitioner Initials (Prescriber)

RN SIGNATURE DATE/TIME

DT0077

MEDICATION ORDER
CYCLOPHOSPHAMIDE
(CYTOXAN®) INFUSION
NON-ONCOLOGY
USE ONLY

Form#0039

STANDARD ADMINISTRATION TIMES

Daily 0800	Nightly 2100	Every 3 hours 0000-0300-0600-0900-1200-1500-1800-2100
Twice a day 0800-2000	Three times a day 0800-1400-2000	Every 4 hours 0000-0400-0800-1200-1600-2000
Every 12 hours 0800-2000	Four times a day 0800-1200-1600-2000	Every 6 hours 0200-0800-1400-2000
		Every 8 hours 0000-0800-1600

OTHER ORDERS

	DESCRIPTION	INSTRUCTIONS
☒	Return for next infusion in ____ weeks	
☒	CBC 14 days after infusion.	
☐		

-------DO NOT WRITE BELOW THIS LINE – IF YOU NEED ADDITIONAL ORDERS, PLEASE USE PHYSICIAN ORDER SHEET-------

Print Name **Ordering Practitioner's Signature** **Date/Time** **Beeper Number**

Print Name **(Co-Signature) Practitioner's Signature** **Date/Time** **Beeper Number**

Attending Co-signature if NP/Resident has ordered drug

Licensed Independent Practitioner Initials (Prescriber) **RN SIGNATURE DATE/TIME**

RHEUMATOLOGY INFLIXIMAB (Remicade) INFUSION ORDERS # ___

**Date:______ / _______ / ________ Time:_____ ________________**		**Dose # ________**	
**MEASUREMENTS**			
**Admit Wt: ______ Kg** ☒Measured		**Ht:_______Cm** ☒Measured	
• Items with boxes must be checked to be ordered<br>• Strike thru must be thru entire line and must be initialed by the physician to be valid.<br>• The following abbreviations CANNOT be used:<br>**cc u IU q.d. q.o.d. MS MS04 MgSO4**		• CANNOT use trailing Zero (X.0 mg) or Leading decimal point (.X mg) (always use a leading zero)<br>• The metric system must be used to enter all medication orders<br>• All pages of order set MUST be sent to Pharmacy	
**ALLERGIES/SENSITIVITIES**			
☐No Known Allergies	☐Latex Allergies	☐Latex Precautions	☐Unable to Obtain
**Agent**		**Reaction(s)/Notes**	
1.			
2.			
**Other:**			

**INDICATION FOR USE OF THIS ORDER SET – Infliximab (Remicade®)**
Indication: ICD 10 -
**ADMINISTRATION RESTRICTIONS/INFORMATION**
**Patient must have negative result of TB skin test on file (within last 12 months) prior to infusion (see Logician)**

**VITALS**				
	**DESCRIPTION**	**FREQUENCY**	**NOTIFY PRESCRIBER**	**INSTRUCTIONS**
☒	Vital Signs<br>☒Pulse<br>☒Respirations<br>☒Temperature	☒Every 10 minutes x 2, then every 30 minutes x 2, then hourly until infusion completes<br>☐Every 15 minutes during infusion<br>☐ ________	☒ Temp greater than 101°F<br>☐ HR greater than____ or less than____<br>☐ RR greater than ____ or less than ___	
☒	Blood Pressure	☒Every 10 minutes x 2, then every 30 minutes x 2, then hourly until infusion completes<br>☐Every 15 minutes during infusion<br>☐ ________	☒ SBP greater than __150__ or less than__90__<br>☒ DBP greater than __90__ or less than__50__	

**NURSING/TREATMENTS**			
	**DESCRIPTION**	**FREQUENCY**	**INSTRUCTIONS**
☐	Intake and Output	☐Strict (Measure) ☐Routine (Count)<br>☐Every ______during infusion	Notify Prescriber if UOP drops below 1 mL/kg/hr averaged over ______ hours

	**Description**	**Instructions**
☐	Notify prescriber	prior to starting infusion
☒	Notify Prescriber	☒ For suspected medication reaction (generalized hives; shortness of breath/wheezing; nausea/vomiting/diarrhea)<br>☐ If patient loses IV access<br>☐ Severe muscle aches or headache
☐	Please schedule next appointment for infusion:	_______ weeks

Licensed Independent Practitioner Initials (Prescriber) **RN SIGNATURE DATE/TIME**

DT0077

MEDICATION ORDER
INFLIXIMAB (REMICADE®)

Form#0047

STANDARD ADMINISTRATION TIMES

Daily 0800	Nightly 2100	Every 3 hours 0000-0300-0600-0900-1200-1500-1800-2100
Twice a day 0800-2000	Three times a day 0800-1400-2000	Every 4 hours 0000-0400-0800-1200-1600-2000
Every 12 hours 0800-2000	Four times a day 0800-1200-1600-2000	Every 6 hours 0200-0800-1400-2000
		Every 8 hours 0000-0800-1600

	VASCULAR ACCESS	INSTRUCTIONS	COMMENTS
☒	Peripheral IV ☒ Insert ☒ Maintain	Saline lock when not in use	
☐	Central Vascular Access	☐ See Central Vascular Access Order Set	

MEDICATIONS FOR VASCULAR ACCESS

	MEDICATION NAME	DOSE	ROUTE	FREQUENCY	INSTRUCTIONS
☒	Lidocaine 4% Cream (LMX)	1 Application (Dispense 1 tube)	Topical-skin (procedure site)	PRN venipuncture (max every 2 hours)	Apply 30 minutes before procedure with occlusive dressing

PRE-MEDICATION ORDERS

	MEDICATION NAME (GENERIC PREFERRED)	DEFAULT (REFERENCE)	DOSE	ROUTE	FREQUENCY	DURATION	PRIORITY	INSTRUCTIONS (IF PRN – INDICATION IS REQUIRED)
☒	Acetaminophen	10 -15 mg/kg/dose (Max: 90 mg/kg/ day; ADULT MAX 4,000 mg/day)		☒ PO ☐ PR	Once			Give 30 minutes prior to infusion
☒	Diphenhydramine	0.5-1 mg/kg/dose (Max: 50mg/dose		☒PO ☐IV	PRN			Give 30 minutes prior to infusion
☐	Hydrocortisone	1-2 mg/kg/dose (Max: 100mg/dose)		IV	Once			
☐	Methylprednisolone (Solumedrol®)	1-2mg/kg/dose (Max: 100mg/dose)		IV	Once			

EMERGENCY MEDICATION ORDERS

	MEDICATION NAME (GENERIC PREFERRED)	DEFAULT (REFERENCE)	DOSE	ROUTE	FREQUENCY	DURATION	PRIORITY	INSTRUCTIONS (IF PRN – INDICATION IS REQUIRED)
☒	Diphenhydramine	0.5-1 mg/kg/dose (Max: 50mg/dose		IV	Once PRN			*** For treatment of anaphylaxis reactions with Infliximab.** **Must notify prescriber****
☒	Epinephrine 1:1000 (EPIPEN® -0.3mg, EPIPEN JR® - 0.15mg) ****Reserved for outpatient use****		☐0.15 mg (10-25 kg) ☐ 0.3 mg (>25 kg)	IM	Once PRN			*** For treatment of anaphylaxis reactions with Infliximab.** **Must notify prescriber****

Licensed Independent Practitioner Initials (Prescriber) ____________

RN SIGNATURE DATE/TIME ____________

DT0077

MEDICATION ORDER
INFLIXIMAB (REMICADE®)

Form#0047

STANDARD ADMINISTRATION TIMES

Daily 0800	Nightly 2100	Every 3 hours 0000-0300-0600-0900-1200-1500-1800-2100
Twice a day 0800-2000	Three times a day 0800-1400-2000	Every 4 hours 0000-0400-0800-1200-1600-2000
Every 12 hours 0800-2000	Four times a day 0800-1200-1600-2000	Every 6 hours 0200-0800-1400-2000
		Every 8 hours 0000-0800-1600

MEDICATION INFUSION ORDERS

	MEDICATION NAME (GENERIC PREFERRED)	DEFAULT (REFERENCE)	DOSE	ROUTE	FREQUENCY	DURATION	PRIORITY	INSTRUCTIONS (IF PRN – INDICATION IS REQUIRED)
☒	Infliximab (Remicade®)	3-20 mg/kg/dose		IV	Once	Infuse over _**2**_hours (normal 2-4 hours)		Begin infusion at 0.1mL/kg/hr and advance to maintenance after 10 minutes **Round to nearest 50mg or 100mg**
☐	**Methylprednisolone Pulse Infusion (Please see appropriate order set)**							

LABS (Drug levels and Coags should not be routinely drawn from central line)

	NAME OF TEST	PRIORITY	FREQUENCY	DURATION	INSTRUCTIONS/PRN INDICATIONS
☒	Complete Blood Count with automated differential	Routine	Once		Obtain prior to start of infusion.
☒	Complete Metabolic Panel	Routine	Once		Obtain prior to start of infusion.
☒	C-reactive Protein (CRP)	Routine	Once		Obtain prior to start of infusion.
☒	Erythrocyte Sedimentation Rate (ESR)	Routine	Once		Obtain prior to start of infusion.
		Routine	Once		Obtain prior to start of infusion.

OTHER ORDERS

	DESCRIPTION	INSTRUCTIONS
☐		
☐		
☐		

Print Name _______________ **Ordering Practitioner's Signature** _______________ **Date/Time** _______________ **Beeper Number** _______________

Licensed Independent Practitioner Initials (Prescriber) _______________ **RN SIGNATURE DATE/TIME** _______________

RHEUMATOLOGY IMMUNE GLOBULIN (IVIg) INFUSION ORDERS # ___

Date:____ / ____ / ________ Time:____________________

MEASUREMENTS

Admit Wt: ______ Kg ☒Measured **Ht:_______Cm** ☐Measured

- Items with boxes must be checked to be ordered
- Strike thru must be thru entire line and must be initialed by the physician to be valid.
- The following abbreviations CANNOT be used:
  cc u IU q.d. q.o.d. MS MS04 MgSO4

- CANNOT use trailing Zero (X.0 mg) or Leading decimal point (.X mg) (always use a leading zero)
- The metric system must be used to enter all medication orders
- All pages of order set MUST be sent to Pharmacy

ALLERGIES/SENSITIVITIES

☐No Known Allergies ☐Latex Allergies ☐Latex Precautions ☐Unable to Obtain

**Agent**	**Reaction(s)/Notes**
1.	
2.	

Other:

INDICATION FOR USE OF THIS ORDER SET – Intravenous Immune Globulin

Indication:

MEDICATIONS --

	MEDICATION NAME (GENERIC PREFERRED)	DEFAULT (REFERENCE)	DOSE	ROUTE	FREQUENCY	DURATION	PRIORITY	INSTRUCTIONS (IF PRN – INDICATION IS REQUIRED)
☒	Acetaminophen	10 -15 mg/kg/dose (Max 650 mg/dose)		PO	☐Once<br>☐ _____			Give 30 minutes prior to infusion<br>☐PRN for IVIG
☒	Diphenhydramine	0.5-1 mg/kg/dose (Max: 50mg)		☐PO<br>☐IV	☐Once<br>☐ _____			Give 30 minutes prior to infusion<br>☐PRN for IVIG
**EMERGENCY MEDICATION ORDERS**								
☒	Diphenhydramine	0.5-1 mg/kg/dose (Max: 50mg)		IV	☐Once PRN<br>☐ ______			*** Epinephrine should be immediately available for treatment of anaphylaxis reactions with IVIG.**<br>☐PRN for IVIG
☒	Epinephrine 1:1000 (***EPIPEN***® - 0.3mg, ***EPIPEN JR***® - 0.15mg) ****Reserved for outpatient use****	0.01 mg/kg/ dose (0.01mL/ kg/ dose) Max dose: 0.5mg or 0.5mL	☐0.15 mg (10-25 kg)<br>☐0.3 mg (>25 kg)	IM	Once PRN			*** Epinephrine should be immediately available for treatment of anaphylaxis reactions with IVIG.**<br>☐PRN for IVIG

Licensed Independent Practitioner Initials (Prescriber) **RN SIGNATURE DATE/TIME**

DT0077

MEDICATION ORDER
IVIG

Form#0027

**STANDARD ADMINISTRATION TIMES**		Every 3 hours 0000-0300-0600-0900-1200-1500-1800-2100
Daily 0800	Nightly 2100	Every 4 hours 0000-0400-0800-1200-1600-2000
Twice a day 0800-2000	Three times a day 0800-1400-2000	Every 6 hours 0200-0800-1400-2000
Every 12 hours 0800-2000	Four times a day 0800-1200-1600-2000	Every 8 hours 0000-0800-1600

MEDICATIONS --								
	**MEDICATION NAME (GENERIC PREFERRED)**	**DEFAULT (REFERENCE)**	**DOSE**	**ROUTE**	**FREQUENCY**	**DURATION**	**PRIORITY**	**INSTRUCTIONS (IF PRN – INDICATION IS REQUIRED)**
☐	Immune Globulin<br>☐Gammagard®<br>☐Gamunex®	**2gm/kg**		IV	☐Once<br>☐ _______			Round to nearest 5 Grams<br>****Please see infusion instructions****
☐	Immune Globulin (**Non-formulary Brand**, must specify)<br>☐PolyGam<br>☐Octagam<br>☐Carimmune NF<br>☐Privigen			IV	☐Once<br>☐ _______			**Contact immunology or pharmacy for appropriate rate.**<br><br>*Non-formulary request must be submitted*
☐	**Methylprednisolone Pulse Infusion (Please see appropriate order set)**							

**INFUSION AND ADMINSTRATION INSTRUCTIONS for IVIG (Please refer to appendix for detailed administration information)**
Begin infusion at ________ ml/hr. (0.6ml/kg/hr) for 15-30 minutes
If no adverse reaction, increase to _______ ml/hr. (1.2 ml/kg/hr) for 15-30 minutes
If no adverse reaction, increase to _______ml/hr.(2.4-ml/kg/hr) for 15-30 minutes
If no adverse reaction, increase to _______ml/hr.(3.6-ml/kg/hr) for 15-30 minutes
If no adverse reaction, increase to _______ml/hr.(4.8-ml/kg/hr) until complete

**OTHER ORDERS**		
	**DESCRIPTION**	**INSTRUCTIONS**
☒	Notify Prescriber	For suspected medication reaction or if patient loses IV access
☒	Return for next infusion in _______weeks	
☒	Labs:	

Appendix to Intravenous Immune Globulin (IVIG) Medication Order Set – This section is to be used as a reference for administration.

1. IVIG solutions should be brought to room temperature before administration: should not be shaken or mixed with any other drugs or blood products.
2. All IVIG brands may be filtered but only Polygram® S/D, Gammagard® S/D, and Iveegam EN require filtration. A 15 micron filter is recommended, but flow may be hindered.
3. Infusion rate should begin at 0.01 ml/kg/minute (0.6ml/kg/hr) for 15 to 30 minutes, then the rate may be increased to 0.02ml/kg/minute (1.2ml/kg/hr). Most patients will then tolerate a gradual increase to 0.04-0.08 ml/kg/minute (2.4-3.6ml/kg/hr).
4. The compatibility of IVIG with IV fluids is product dependent. Contact Pharmacy for information on specific brands.
5. Adverse reactions are generally rate related and may be eliminated by decreasing the rate or stopping the infusion and/or giving acetaminophen, aspirin, and diphenhydramine.

**Adverse reactions include:**

URTICARIA FEVER FLUSHING FATIGUE MUSCULAR PAIN HEADACHE CHILLS WHEEZING DYSPNEA * POSSIBLY ANAPHYLAXIS

-------DO NOT WRITE BELOW THIS LINE – IF YOU NEED ADDITIONAL ORDERS, PLEASE USE PHYSICIAN ORDER SHEET-----________________________________

Print Name ______________________________ **Ordering Practitioner's Signature** __ ____________ **Date/Time** _____________ **Beeper Number**____ ____________________________________

Licensed Independent Practitioner Initials (Prescriber) **RN SIGNATURE DATE/TIME**

RHEUMATOLOGY METHYLPREDNISOLONE (Solumedrol) INFUSION ORDERS # ___

Date:_____/ _______/ ______ Time:_____1300_______________

MEASUREMENTS

Admit Wt: _______ Kg ☒Measured **Ht:_______Cm** ☒Measured

- Items with boxes must be checked to be ordered
- Strike thru must be thru entire line and must be initialed by the physician to be valid.
- The following abbreviations CANNOT be used: **cc u IU q.d. q.o.d. MS MS04 MgSO4**
- CANNOT use trailing Zero (X.0 mg) or Leading decimal point (.X mg) (always use a leading zero)
- The metric system must be used to enter all medication orders
- All pages of order set MUST be sent to Pharmacy

ALLERGIES/SENSITIVITIES

☐No Known Allergies ☐Latex Allergies ☐Latex Precautions ☐Unable to Obtain

**Agent**	**Reaction(s)/Notes**
1.	
2.	

Other:

INDICATION FOR USE OF THIS ORDER SET – METHYLPREDNISOLONE (SOLU-MEDROL®) PULSE DOSE INFUSION

Indication (required): ICD10 -

NURSING ORDERS

	**DESCRIPTION**	**INSTRUCTIONS**
☒	Notify Prescriber	For suspected medication reaction if patient loses IV access, or any acute changes in patient's condition

	**VASCULAR ACCESS**	**INSTRUCTIONS**	**COMMENTS**
☒	Peripheral IV ☒ Insert ☒ Maintain	Saline lock when not in use	
☐	**See Supportive Care Central Line (Excluding Dialysis) Order Set**		

MEDICATIONS FOR VASCULAR ACCESS

	**MEDICATION NAME**	**DOSE**	**ROUTE**	**FREQUENCY**	**INSTRUCTIONS**
☒	Lidocaine 4% Cream (LMX)	1 Application (Dispense 1 tube)	Topical-skin (procedure site)	PRN venipencture (max every 2 hours)	Apply 30 minutes before procedure with occlusive dressing

MEDICATION ORDERS

	**MEDICATION NAME (GENERIC PREFERRED)**	**DEFAULT (REFERENCE)**	**DOSE**	**ROUTE (PICK ONE)**	**FREQUENCY**	**DURATION**	**PRIORITY**	**INSTRUCTIONS**
☒	Methylprednisolone	30 mg/kg/dose (Max: 1000 mg)	**1000mg**	IV	Once Daily for_____ doses			Infuse over 1 hour

OTHER ORDERS

	**DESCRIPTION**	**INSTRUCTIONS**
☒	Please schedule next clinic appointment for infusion	_________ week(s)

-------DO NOT WRITE BELOW THIS LINE – IF YOU NEED ADDITIONAL ORDERS, PLEASE USE PHYSICIAN ORDER SHEET-------

Print Name ________________________ **Ordering Practitioner's Signature** ____________________________ **Date/Time** _______________ **Beeper Number** ______________

____ **Licensed Independent Practitioner Initials (Prescriber)**

____________________________________ **RN SIGNATURE DATE/TIME**

RHEUMATOLOGY N-Acetylcysteine INFUSION ORDERS # ___

1. Attending MD: _________________
2. Diagnosis: ____Crest syndrome (ICD9-710.9)_______________________
3. Weight:__51.1__kg Height:__141.8__cm
4. Vital Signs q15 min X 4, then q30 min X 2, then qh.
5. Notify MD for: SBP less than __90___or greater than ___150__
   DBP less than __50___ or greater than __90___

6. Medications:

- N-Acetylcysteine ________ mg (__15mg/kg/hr__) IV
  diluted in 500ml of D5 ½ NS to run over 5 hours (100ml/hr).

- Return for next infusion in _____ week/s.

Signature ____
Date/Time: ___________________

RHEUMATOLOGY PAMIDRONATE INFUSION # ___

Attending MD: _______________

1. Diagnosis: ____________________
2. Vital signs and weight prior to infusion; Temp. and **BP** every 30 minutes during infusion
3. **Labs:** Ionized calcium, osteocalcin, alkaline phosphatase,
   ** Ionized calcium level at end of infusion
4. IV to Hep lock. LMX cream or equivalent prior to insertion PRN.
5. Diet: regular
6. During infusion, patient may be up only to void.
7. **Medication: Pamidronate** _____ _____ (1.5mg/kg mixed with NS, max 90mg) and infuse over 4 hours.
8. .Notify MD for Adverse reactions including:
   Altered vital signs, difficulty breathing, HR > 100, Temp. > 101
9. Patient may be discharged home after infusion completed, **must have normal calcium level verified by Attending MD and vital signs stable.**
   **** Notify Attending MD when infusion completed and Calcium level post infusion drawn.**

- Return for next infusion in ___3___ months for Pamidronate # _____.

Signature ____________________________ /_________________________
Date/Time: _______________

RHEUMATOLOGY RITUXIMAB (Rituxan) INFUSION ORDERS # ___

Date:____ / ______ / ________ **Time:**____________________ **Dose #**____ **Round #**____

MEASUREMENTS

Admit Wt: ______ **Kg** ☒Measured **Ht:**_______**Cm** ☒Measured **BSA (m2):** ________

- Items with boxes must be checked to be ordered
- Strike thru must be thru entire line and must be initialed by the physician to be valid.
- The following abbreviations CANNOT be used: **cc u IU q.d. q.o.d. MS MS04 MgSO4**
- CANNOT use trailing Zero (X.0 mg) or Leading decimal point (.X mg) (always use a leading zero)
- The metric system must be used to enter all medication orders
- All pages of order set MUST be sent to Pharmacy

ALLERGIES/SENSITIVITIES

☐No Known Allergies ☐Latex Allergies ☐Latex Precautions ☐Unable to Obtain

**Agent**	**Reaction(s)/Notes**
1.	
2.	
**Other:**	

INDICATION FOR USE OF THIS ORDER SET – Rituximab (Rituxan®)

Indication: ICD 10 -

PRESCRIBER RESTRICTIONS/INFORMATION

Labs to be drawn prior to start of infusion

VITALS

	DESCRIPTION	FREQUENCY	NOTIFY PRESCRIBER	INSTRUCTIONS
☒	Vital Signs (Pulse, Respirations)	☒Every 15 minutes x 4, then every hour during infusion<br>☐ ________	☐ HR greater than ____ or less than _____<br>☐ RR greater than ____ or less than _____	
☒	Temperature	☒Every 15 minutes x 4, then every hour during infusion<br>☐ ________	☐ Temp less than ____<br>☒ Temp greater than _101__	
☒	Blood Pressure	☒Every 15 minutes x 4, then every hour during infusion<br>☐ ________	☒ SBP greater than _150____or less than _90___<br>☒ DBP greater than _90____or less than _50___	

NURSING ORDERS

	DESCRIPTION	INSTRUCTIONS
☒	Notify Prescriber	For suspected medication reaction or if patient loses IV access
☒	Please schedule next clinic appointment for infusion	__________ week(s)

	VASCULAR ACCESS	INSTRUCTIONS	COMMENTS
☒	Peripheral IV ☒Insert ☒ Maintain	Saline lock when not in use	
☐	**See Supportive Care Central Line (Excluding Dialysis) Order Set**		

MEDICATIONS FOR VASCULAR ACCESS

	MEDICATION NAME	DOSE	ROUTE	FREQUENCY	INSTRUCTIONS
☒	Lidocaine 4% Cream (LMX)	1 Application (Dispense 1 tube)	Topical-skin (procedure site)	PRN venipuncture (max every 2 hours)	Apply 30 minutes before procedure with occlusive dressing

Licensed Independent Practitioner Initials (Prescriber) **RN SIGNATURE DATE/TIME**

DT0077

MEDICATION ORDER

RITUXIMAB
(RITUXAN®) INFUSION ALL

Form#0040

STANDARD ADMINISTRATION TIMES

Daily 0800 | Nightly 2100
Twice a day 0800-2000 | Three times a day 0800-1400-2000
Every 12 hours 0800-2000 | Four times a day 0800-1200-1600-2000
Every 3 hours 0000-0300-0600-0900-1200-1500-1800-2100
Every 4 hours 0000-0400-0800-1200-1600-2000
Every 6 hours 0200-0800-1400-2000
Every 8 hours 0000-0800-1600

	MEDICATION NAME (GENERIC PREFERRED)	DEFAULT (REFERENCE)	DOSE	ROUTE	FREQUENCY	DURATION	PRIORITY	INSTRUCTIONS (IF PRN – INDICATION IS REQUIRED)
**PRE-MEDICATION ORDERS**								
☐	Methylprednisolone (Solumedrol®)	1-2mg/kg/dose (Max: 100 mg/dose)		IV	Once			Give 30 minutes prior to infusion **Not for use as high-dose pulse infusion**
☒	Acetaminophen	10-15 mg/kg/dose (Max: 650 mg/dose)		PO	Once			Give 30 minutes prior to infusion
☒	Diphenhydramine	0.5-1 mg/kg/dose (Max: 50 mg/dose)		☒PO ☐IV	Once			Give 30 minutes prior to infusion
☐	**See Methylprednisolone Pulse Infusion Order Set – if applicable**							
**EMERGENCY MEDICATION ORDERS**								
☒	Epinephrine 1:1,000	0.01 mg/kg/ dose (0.01 mL/kg/dose) Max dose: 0.5 mg or 0.5 mL		IM	Once PRN			*** For treatment of anaphylaxis reactions with Rituximab **Must notify prescriber****
☒	Epinephrine 1:1000 (**EPIPEN® - 0.3mg, EPIPEN JR® - 0.15mg**) ****Reserved for outpatient use****		☐0.15 mg (10-25 kg) ☐0.3 mg (Greater than 25 kg)	IM	Once PRN			*** For treatment of anaphylaxis reactions with Rituximab **Must notify prescriber****
☒	Diphenhydramine	0.5-1 mg/kg/dose (Max: 50 mg/dose)		☒IV ☐IM	Once PRN			*** For treatment of anaphylaxis reactions with Rituximab.**
☒	Hydrocortisone	2 mg/kg/dose (Max: 150 mg/dose)		IV	Once PRN			*** For treatment of anaphylaxis reactions with Rituximab.**
**RITUXIMAB INFUSION**								
☒	Rituximab (Rituxan®)	☐375mg/m^2 ☒750mg/m^2 (Max: 1000mg/dose)		IV	Once			Pharmacy note: Dilute in NS (Max conc: 4mg/ml) *****See Below for Infusion Instructions. *****

☐	**INFUSION AND ADMINSTRATION INSTRUCTIONS**
Start infusion: 0.25mL/kg/hr = _____mL/hr (max of 25mL/hr) for 1st hour	
**If no hypersensitivity:** Increase infusion by increments of 0.25mL/kg/hr = _____mL/hr (max increase of 25mL/hr) every 30 minutes to	

final infusion rate of 100mL/hr
For hypersensitivity event, stop infusion and notify prescriber
**Stop infusion immediately for bronchospasm or hypotension and notify prescriber**
Upon improvement of hypersensitivity symptoms, prescriber may resume infusion at 50% of previous rate

DT0077

MEDICATION ORDER
RITUXIMAB
(RITUXAN®) INFUSION ALL

Form#0040

STANDARD ADMINISTRATION TIMES
Daily 0800 Nightly 2100
Twice a day 0800-2000 Three times a day 0800-1400-2000
Every 12 hours 0800-2000 Four times a day 0800-1200-1600-2000
Every 3 hours 0000-0300-0600-0900-1200-1500-1800-2100
Every 4 hours 0000-0400-0800-1200-1600-2000
Every 6 hours 0200-0800-1400-2000
Every 8 hours 0000-0800-1600

LABS

	NAME OF TEST	PRIORITY	FREQUENCY	DURATION	INSTRUCTIONS/PRN INDICATIONS
☒	Complete Blood Count with Differential	Routine	Once		Obtain prior to start of infusion.
☒	Comprehensive Metabolic Panel	Routine	Once		Obtain prior to start of infusion.
☒	C-Reactive Protein	Routine	Once		Obtain prior to start of infusion.
☒	Erythrocyte Sedimentation Rate	Routine	Once		Obtain prior to start of infusion.
☒	CD 19/20 (B-cell lymphocyte subtype level)	Routine	Once		Obtain prior to start of infusion.

OTHER ORDERS

	DESCRIPTION	INSTRUCTIONS
☐		
☐		

-------DO NOT WRITE BELOW THIS LINE – IF YOU NEED ADDITIONAL ORDERS, PLEASE USE PHYSICIAN ORDER SHEET-------

Print Name **Ordering Practitioner's Signature** **Date/Time** **Beeper Number**
